# Optimization of processing technology and analysis of aroma components of osmanthus black tea using summer and autumn tea leaves

**DOI:** 10.1016/j.fochx.2026.103511

**Published:** 2026-01-15

**Authors:** Haomu Liao, Xiaoyue Song, Yuqin Xiong, Chunhua Ma, Hetong Lin

**Affiliations:** a College of Tea and Food Science, Wuyi University, Wuyishan, Fujian 354300, China; b College of Food Science, Fujian Agriculture and Forestry University, Fuzhou, Fujian 350002, China

**Keywords:** Summer and autumn tea, Osmanthus black tea, Process optimization, Aroma, Quality

## Abstract

Tea is mainly produced with spring tea leaves. Summer and autumn tea leaves, characterized by a bitter and astringent taste, are usually discarded. Here, they were turned into osmanthus black tea, and the processing technology was optimized. Optimum process: the ratio of osmanthus to tea was 1:5.3, 16.6 h scenting, two cycles, the highest sensory score was 92.2. *E*-nose showed significant differences in response values among the W5S, W1W, and W2S sensors. Samples 6, 9, 11, and 12 exhibited high response values across multiple sensors. GC–MS was used to analyze volatile components in osmanthus black tea and the black tea base. A total of 259 volatile components were identified, and 14 key aroma-active compounds were screened, including β-ionone, γ-dodecalactone, γ-decalactone, phenylethanol, and linalool.

## Highlights

• Optimized osmanthus black tea processing from summer-autumn leaves, with top sensory score 92.2 under specific conditions.
• 259 volatile components were identified by gas chromatography–mass spectrometry, and 14 key aroma-active components were screened out.
• Osmanthus black tea had ∼2× more alcohols/ketones, half the esters vs black tea base, higher total aroma.

## 1 Introduction

Tea, one of the most popular beverages worldwide, is rich in bioactive substances such as tea polyphenols, caffeine, amino acids, vitamins, and minerals (Yin et al., 2022). It exhibits various health benefits, including weight loss, reducing blood pressure, blood lipids, and blood sugar, antioxidant activity, anti-inflammatory and antibacterial properties, and alleviation of neurodegenerative diseases (Chen, Chen, et al., 2020; Chen, Liu, et al., 2022; Y. Chen et al., 2013). Tea leaves are primarily harvested and processed in spring. During summer and autumn, high temperatures accelerate tea plant growth, leading to excessive accumulation of tea polyphenols, resulting in a bitter and astringent taste and reduced tea quality (H. Liu et al., 2021), making them rarely utilized (Liang et al., 2024), which is a waste of resources. Recent study has shown that addition of cellulase, polyphenol oxidase, and peroxidase during rolling and fermentation significantly increased the contents of free amino acids, freshness index, total theaflavins, and thearubigins, while enhancing the antioxidant and metabolic capacities of black tea (Chiang et al., 2022). For low-grade oolong tea made from summer and autumn tea leaves, tannase treatment of the final product reduced astringency, enhanced freshness, improved tea quality, and increased storage stability. This effect was consistent with that of tannase on green tea (Cao et al., 2019).

Flower tea is a kind of reprocessed tea produced by mixing tea bases with flowers and scenting to integrate the aromas of tea and flowers (Li et al., 2022). It is typically made from green tea, black tea, or oolong tea scented with jasmine, rose, osmanthus, etc. (Shen et al., 2020). Jasmine tea dominates China's flower tea market (Liu, 2021), while osmanthus is a traditional Chinese food (Wu et al., 2022), with rich fragrance and health benefits such as anti-inflammatory, anti-tumor, antioxidant, blood sugar-lowering, and lipid-lowering effects (B. Huang et al., 2015; S. Huang et al., 2023). There are many varieties of osmanthus, such as gold osmanthus, silver Osmanthus and red Osmanthus. Red osmanthus was used to scent black tea, and the scented tea was found to exhibit a distinct osmanthus aroma (Huang et al., 2022).

In this study, summer and autumn tea leaves was used to produce black tea. Black tea is fully fermented tea (Chen, Zhu, et al., 2022), during fermentation, tea polyphenols are oxidized into theaflavins and thearubigins, reducing the bitter and astringent taste (Zhang, Fu, et al., 2021). The black tea then was scented with osmanthus to incorporate its fragrance. This research investigated the processing technology and aroma components of osmanthus black tea made from summer and autumn tea leaves, hope to provide technical and theoretical support for the utilization of summer and autumn tea resources.

## 2 Materials and methods

### 2.1 Samples

The black tea base was prepared from Huangguanyin cultivar leaves harvested in October 2023, following the standard of one bud with three leaves, using traditional black tea processing techniques. It was provided by Xuling Tea Factory in Wuyishan City, Fujian Province. The osmanthus variety was Dangui, grown on the campus of Wuyi University, and harvested 2 days after blooming. The scenting process included: flower treatment, tea base treatment, mixing of flowers and tea, aeration and continued scenting, drying with flowers, flower removal, and blending and packaging (Cheng et al., 2018).

### 2.2 Materials and instruments

Ethyl decanoate (chromatographic grade) was purchased from Macklin Biochemical Co., Ltd. (Shanghai, China). The electronic nose PEN3 was from Airsense Analytics (Germany); the 50/30 μm DVB/CAR/PDMS extraction fiber was from Supelco (USA); and the gas chromatography–mass spectrometry (GC–MS) system Q8050NX was from Shimadzu (Japan).

### 2.3 Single-factor and response surface methodology for process optimization

#### 2.3.1 Production process

The processing flow chart is shown below.

**Figure:**
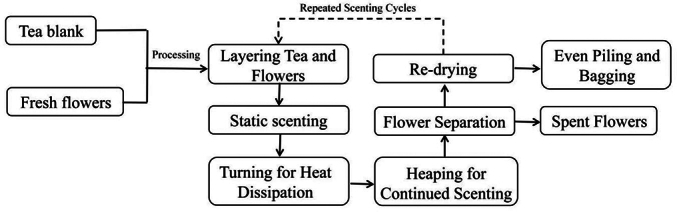

#### 2.3.2 Single-factor experiments

Single-factor experiments were designed using 200 g of processed black tea base per sample. The investigated factors included re-drying time (20, 25, 30, 35, 40 min); re-drying temperature (70, 75, 80, 85, 90 °C); flower-to-tea ratio (1,3, 1:5, 1:7, 1:9, 1:11); scenting time (6, 12, 18, 24, 30 h); and number of scenting cycles (1, 2, 3, 4, 5). For each factor, other parameters were fixed as follows: re-drying time 30 min, re-drying temperature 80 °C, flower-to-tea ratio 1:5, scenting time 18 h, and 2 scenting cycles. Each treatment was repeated 3 times, and sensory scores were averaged.

#### 2.3.3 Response surface optimization

Based on single-factor results, three factors significantly affecting sensory quality were selected: flower-to-tea ratio, scenting time, and number of scenting cycles. Using Design-Expert 13 software, a 3-factor, 3-level Box-Behnken design was employed with sensory score as the response value. This experiment involves three factors. The flower – to - tea ratio (Factor A) has levels of 1:9 (−1), 1:7 (0), and 1:5 (1); scenting cycles (Factor B) include 1 cycle (−1), 2 cycles (0), and 3 cycles (1); scenting time (Factor C) covers 12 h (−1), 18 h (0), and 24 h (1).

#### 2.3.4 Sensory evaluation method

Sensory evaluation was conducted by a panel of 8 trained panelists (4 males and 4 females, aged 25–50 years), who had received systematic training on tea sensory evaluation (including aroma, taste, and appearance assessment) in accordance with GB/T 23776–2018 and passed the qualification assessment. The evaluation was performed following the criteria specified in GB/T 23776–2018 (General Administration of Quality Supervision, Inspection and Quarantine of the People's Republic of China (AQSIQ), and Standardization Administration of China (SAC), 2018).

### 2.4 Electronic nose detection method

1) Pretreatment: Tea samples were ground and sieved through a 0.2 mm mesh. 0.2 g (accurate to 0.001 g) of tea powder was weighed into a headspace vial, added with 10 mL boiling water, shaken, and allowed to stand for 30 min for electronic nose analysis.

2) Analysis conditions: Gas flow rate 0.4 L/min; cleaning time 200 s; sample collection time 90 s. Each sample was analyzed in triplicate. Information on the electronic nose sensor array is shown in Table S1.

### 2.5 HS-SPME-GC–MS analysis of aroma components

Headspace solid-phase microextraction (HS-SPME) combined with GC–MS was used to extract and separate volatile compounds from tea samples.

Extraction method: Tea samples were ground and sieved through a 60-mesh sieve. 0.1 g (accurate to 0.001 g) of tea powder was weighed into a 20 mL headspace vial, added with 5 mL boiling water, 10 μL ethyl decanoate (10 μg/mL), and a magnetic stir bar. The vial was equilibrated at 75 °C and 250 rpm for 10 min. A 50/30 μm DVB/CAR/PDMS fiber was inserted for headspace extraction for 50 min. Desorption was performed at 250 °C in the injection port for 3 min. Each sample was analyzed in triplicate.

#### 2.5.1 Chromatographic and mass spectrometric conditions

Chromatographic conditions: HP-5MS capillary column (30 m × 250 μm × 0.25 μm); splitless injection; inlet temperature 250 °C; carrier gas flow rate 1.0 mL/min. Temperature program: initial 50 °C (held for 2 min), increased to 80 °C at 2 °C/min (held for 1 min), then to 120 °C at 5 °C/min (held for 1 min), to 140 °C at 4 °C/min (held for 2 min), to 150 °C at 4 °C/min (held for 3 min), to 160 °C at 5 °C/min (held for 2 min), to 180 °C at 10 °C/min (held for 2 min), and finally to 220 °C at 10 °C/min (held for 10 min).

Mass spectrometric conditions: Electron ionization (EI) source; electron energy 70 eV; transfer line temperature 280 °C; ion source temperature 230 °C; quadrupole temperature 150 °C; full scan mode (*m*/*z* 45–600).

### 2.6 Data processing and analysis

Electronic nose data were analyzed using Winmuster software. For GC–MS data, volatile compounds were identified by comparing with the NIST20 database, retention indices, and similarity. Qualification was performed using the internal standard method. SPSS 27 was used for data standardization, and SIMCA 14.1 was used for principal component analysis (PCA), orthogonal partial least squares-discriminant analysis (OPLS-DA), variable importance in projection (VIP) calculation, and fold change (FC) analysis. Analysis of variance (ANOVA) was performed using SPSS, and heatmaps were generated using Origin. All the determination were performed in triplicate (*n* = 3).

## 3 Results and discussion

### 3.1 Single-factor experimental results

**Fig. 1:**
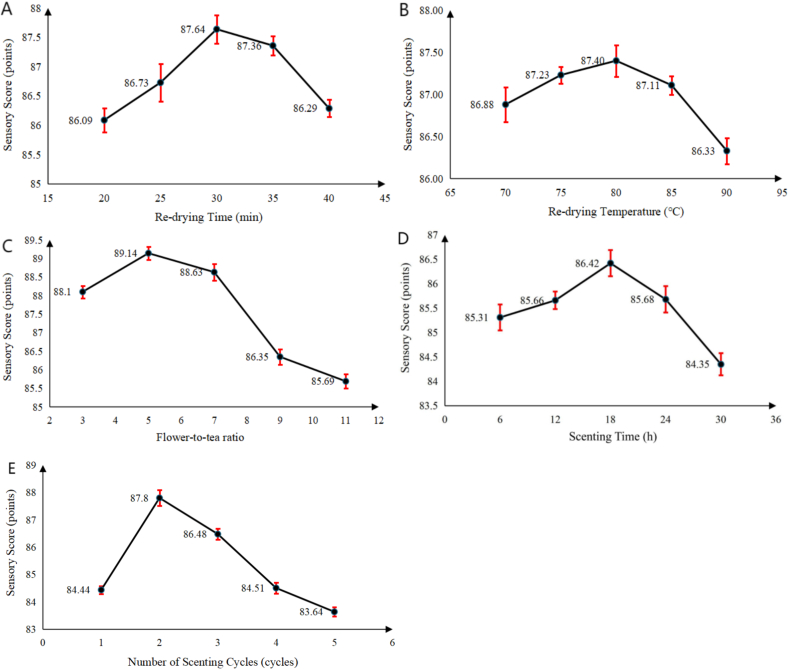
Effect of re-drying time (A) re-drying temperature (B) flower-to-tea ratio (C) scenting time (D) and number of scenting cycles (E) on sensory score.

*Re*-drying maintains tea dryness to stabilize quality, is a key step in osmanthus black tea production. As shown in Fig. 1A, sensory scores initially increased and then decreased as re-drying time prolongs to 30 min, the highest score was 87.64. There were very few differences among groups, thus 30 min was selected for subsequent experiments.

Fig. 1B shows that the highest sensory scores achieved at 87.40 at 80 °C, so 80 °C was chosen for the follow-up experiments.

Scenting integrates black tea aroma with osmanthus fragrance. An appropriate flower-to-tea ratio can balances the mellow taste of black tea and the sweet floral aroma of osmanthus. Fig. 1C shows scores peaking was 89.10 with a 1:5 flower-to-tea ratio. The scenting process is crucial for the harmonious integration of the aroma of *Osmanthus fragrans* and black tea. Through an appropriate scenting time, black tea can fully absorb the fragrance of *Osmanthus fragrans*, resulting in a harmonious blend of the two scents. In Fig. 1D, the sensory score initially increased and then decreased as the scenting time extension. When the scenting time reached 18 h, the highest sensory score was 86.42. Therefore, the optimal scenting time for this experiment is determined to be 18 h.

Multiple scenting rounds contribute to better absorption of the Osmanthus fragrance by black tea leaves, leading to a harmonious flavor combination. During the repeated scenting process, the quality of the tea gradually stabilizes, with a reduction in bitterness and a boost in sweetness. In Fig. 1E, the sensory score shows a trend of initially increasing and then decreasing with the growth of the number of scenting rounds. The highest sensory score was 87.8 achieved when the number of scenting rounds was 2.

### 3.2 Response surface experimental results

#### 3.2.1 Model establishment and data analysis

Based on single-factor results, Design-Expert 13 was used to optimize flower-to-tea ratio, number of scenting cycles, and scenting time. The experimental design and results are shown in Table 1, with the quadratic regression equation:

$$Y=91.80–0.5687\;A-0.6925B+0.5138C+0.8525\mathrm{AB}-0.79\;\mathrm{AC}-0.2225\;\mathrm{BCE}-2.{\text{4A}}^{2}–2.05B^{2}–2.{\text{9C}}^{2}$$

where Y = sensory score, A = flower-to-tea ratio, B = scenting time, and C = number of scenting cycles.

**Table 1:** Box-Behnken design with experimental.

No.	A Flower-to-tea ratio	B scenting time (h)	C number of scenting cycles (cycles)	Sensory score
1	1:5	12	2	89.45
2	1:9	12	2	86.47
3	1:5	24	2	86.54
4	1:9	24	2	86.97
5	1:5	18	1	85.87
6	1:9	18	1	86.45
7	1:5	18	3	88.14
8	1:9	18	3	85.56
9	1:7	12	1	86.73
10	1:7	24	1	85.61
11	1:7	12	3	88.54
12	1:7	24	3	86.53
13	1:7	18	2	92.43
14	1:7	18	2	91.26
15	1:7	18	2	91.35
16	1:7	18	2	92.16
17	1:7	18	2	91.81

ANOVA results (Table 2) showed the model was highly significant (*P* < 0.0001) with a non-significant lack of fit (*P* = 0.742 > 0.05). The coefficient of determination R^2^ = 0.9867, indicating the model explained 98.67% of response variance. R^2^Adj = 0.9696 and R^2^Pre = 0.9323 differed by <0.2, confirming good model fit and reliability for optimizing osmanthus black tea processing.

**Table 2:** Regression model variance analysis. Note: ^⁎^ and ^⁎⁎^ represented the statistical significances at *P* < 0.05 and *P* < 0.01, respectively.

	Sum of Squares	Degrees of Freedom	Mean Square	F-value	P-value	Significance
Model	100.24	9	11.14	57.71	<0.0001	**
A Flower-to-tea ratio	2.59	1	2.59	13.41	0.0081	**
B Scenting time	3.84	1	3.84	19.88	0.0029	**
C Number of scenting cycles	2.11	1	2.11	10.94	0.013	*
AB	2.91	1	2.91	15.06	0.006	**
AC	2.5	1	2.5	12.93	0.0088	**
BC	0.198	1	0.198	1.03	0.3448	
A^2^	24.17	1	24.17	125.23	<0.0001	**
B^2^	17.67	1	17.67	91.54	<0.0001	**
C^2^	35.43	1	35.43	183.59	<0.0001	**
Residual	1.35	7	0.193			
Lack of Fit	0.3304	3	0.1101	0.4316	0.742	Not Significant
Pure Error	1.02	4	0.2552			
Total Sum	101.6	16				

*P*-values indicated flower-to-tea ratio (A), scenting time (B), interactions AB, AC, and BC, and quadratic terms A^2^, B^2^, and C^2^ had highly significant effects (*P* < 0.01); number of scenting cycles (C) had a significant effect (*P* < 0.05). F-values showed the order of influence was B (scenting time) > A (flower-to-tea ratio) > C (number of scenting cycles).

#### 3.2.2 Response surface interaction analysis

**Fig. 2:**
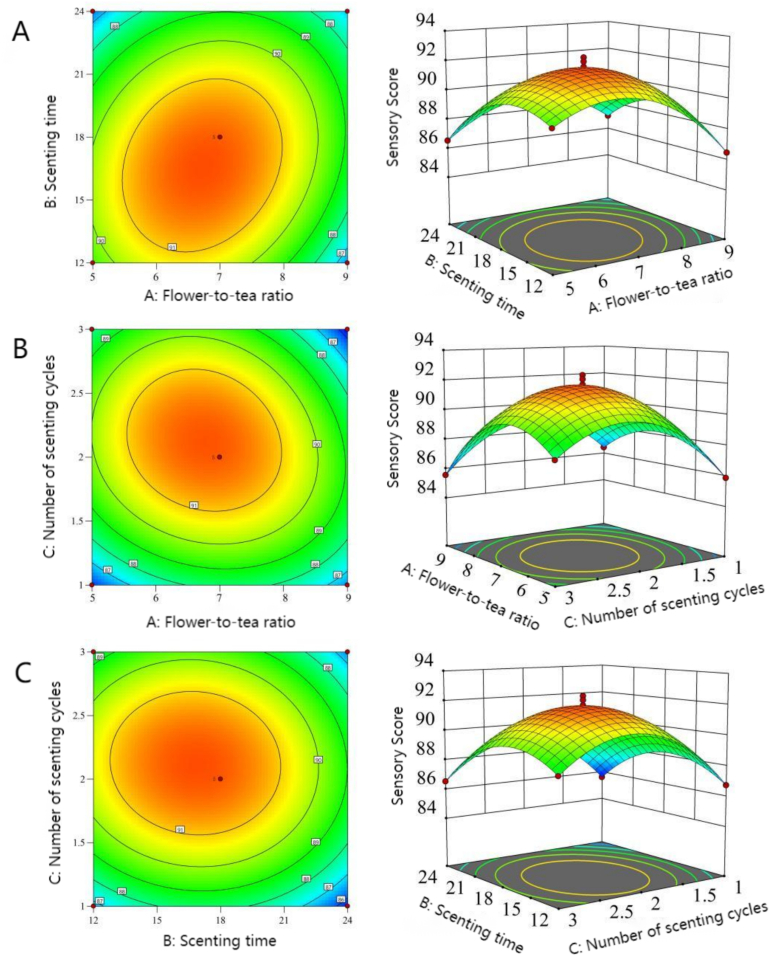
Response surface and contour map of sensory evaluation, (A) camellia ratio and scenting time, (B) camellia ratio and scenting time, (C) scenting time and scenting time.

In the interaction analysis of response surface, the steeper the response surface curve, the stronger the interaction. For the overall model, *P* < 0.0001, which is extremely significant, indicating that the model can effectively explain the variation of sensory scores. Fig. 2A shows that the AB curve has an elliptical contour, indicating that the interaction between the flower - to - tea ratio and the scenting time is significant and jointly affects the sensory score. Secondly, as shown in Fig. 2B, both the ellipticity of the contour lines and the slope of the surface show that the interaction between the flower - to - tea ratio and the number of scenting cycles has a significant impact on the sensory score. However, Fig. 2C shows that the BC curve has a nearly circular contour, indicating that the interaction between the scenting time and the number of scenting cycles is weak and has no significant impact on the sensory score, which is consistent with the P - value in Table 2. According to the analysis of Table 2 and Fig. 2, among the factors, the scenting time (B) has the most significant impact (with the largest F - value), followed by the flower - to - tea ratio (A) and the number of scenting cycles (C).

#### 3.2.3 Verification experiment

The validated optimum parameters derived from response surface optimization were as follows: flower-to-tea ratio of 1:5.31, a scenting time of 16.59 h, and 2.15 cycles, with a predicted sensory score of 91.99. For practical feasibility, conditions were adjusted to 1:5.3, 16.6 h, and 2 cycles. Triplicate verification experiments yielded an average score of 92.18, consistent with the predicted value, confirming model accuracy.

### 3.3 Analysis of Osmanthus black tea with different scenting processes using electronic nose

**Fig. 3:**
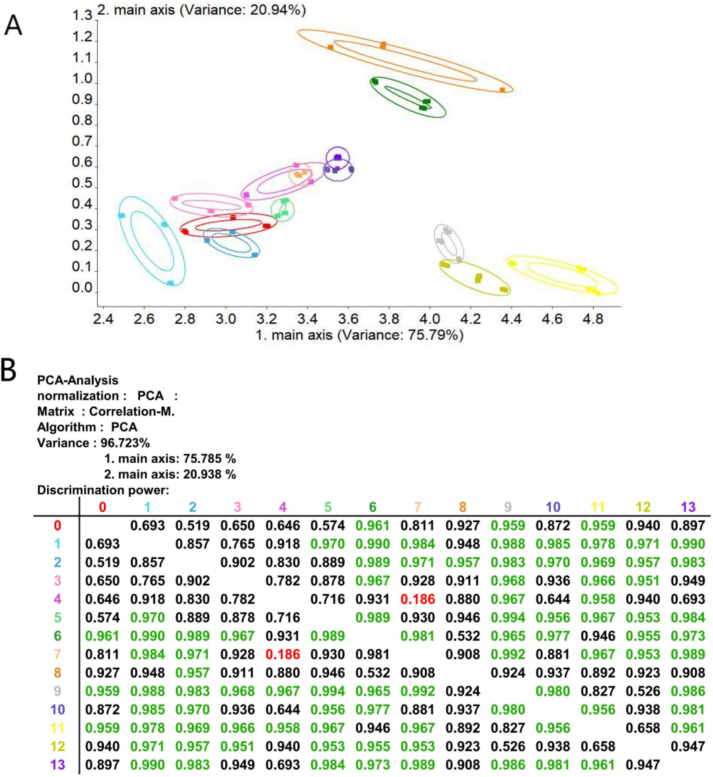
Electronic nose PCA (A) and Correlation (B) of osmanthus black tea with different scenting processes.

PCA was performed on *E*-nose data from osmanthus black tea samples (Fig. 3a). The cumulative variance contribution of PC1 (78.03%) and PC2 (18.95%) reached 96.99%, capturing the primary information characteristics of the samples (Guo, Adelina, et al., 2022). As shown in Fig. 3a, the aroma profiles of Samples 1, 6, 8, and 11 exhibited complete dispersion without overlap with other samples. While the raw tea base showed certain similarity with Samples 2, 3, and 5, significant differentiation (Mahalanobis distance >3.0) was observed for others. Notably, Sample 7 was entirely encompassed by Sample 4, indicating indistinguishable aromatic compositions between these two.

Samples 6 (1:9 FTR, 18 h, 1 cycle) and 8 (1:9 FTR, 18 h, 3 cycles) demonstrated maximal dispersion, likely attributable to distinct volatile substances generated under minimal flower-to-tea ratio combined with 18 h time. This aligns with Table 2 data where FTR × Scenting time interaction showed the strongest effect. Specific differential aroma compounds require further GC–MS investigation.

Discriminability quantification (Fig. 3b) revealed a low correlation (*r* = 0.186, *p* > 0.05) between Sample 4 (1:9 FTR, 24 h, 2 cycles) and Sample 7 (1:5 FTR, 18 h, 3 cycles), confirming aromatic similarity. Compared to the raw tea base, pronounced discriminability (*r* < 0.3) was observed only against Samples 6, 9 (1:7 FTR, 12 h, 1 cycle), and 11 (1:7 FTR, 12 h, 3 cycles). This implies that moderate FTR with minimal time or minimal FTR with moderate time exerts the greatest impact on aroma modulation.

The divergence between Samples 9 and 11 primarily stems from scenting cycle variations. Increased cycles augmented volatile diversity: experimental data showed aroma compound counts rising from 44 to 61 when cycles increased from 1 to 5 (Zhang, Ohland, et al., 2021). However, tea's aroma absorption capacity approaches saturation beyond 3 cycles. Excessive cycles (>4) may trigger volatile exsorption, causing loss of aromas. Moreover, repeated pre-cycle drying induces leaf structural damage, while prolonged thermal exposure accelerates enzymatic browning, leading to undesirable discoloration.

**Fig. 4:**
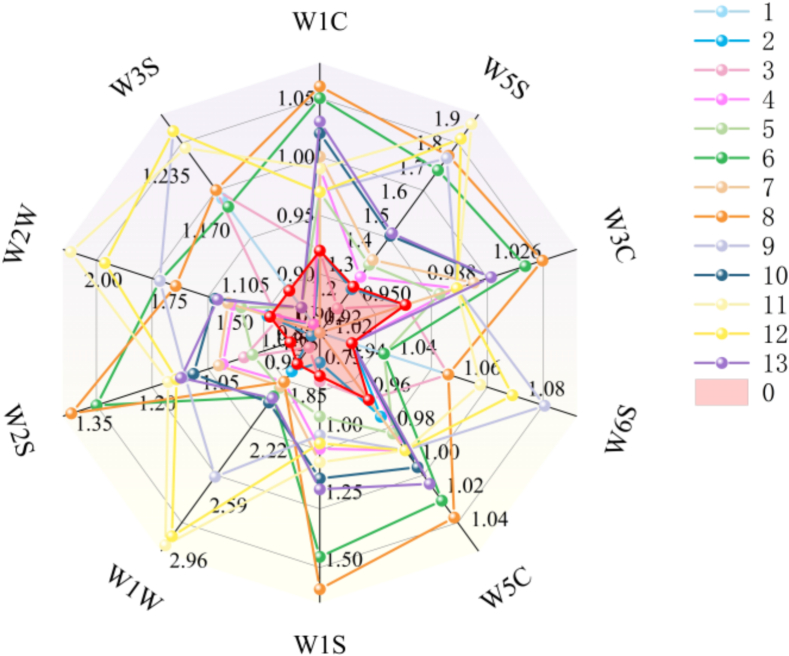
Radar diagram of response value of electronic nose sensor for osmanthus black tea with different scenting processes.

As shown in Fig. 4, sensors W5S, W1W, and W2W exhibited higher response values to tea aromas. The NOₓ-sensitive W5S sensor showed responses ranging from 1.08 to 1.94 for osmanthus black teas, compared to 1.27 for the raw black tea as control. Samples 1, 2, and 3 displayed responses lower than the control, while Sample 4 (1:9 FTR, 24 h, 2 cycles) registered 1.31, closely matching the control. This indicates that 2 scenting cycles reduced nitrogen oxide volatiles – relatively unstable compounds prone to degradation during repeated processing. Competitive adsorption likely occurred, where preferentially-absorbed aromatics occupied binding sites, diminishing NOₓ retention. The elevated NOₓ in 3-cycle teas (e.g., Sample 8) may result from liberated adsorption sites after volatile desorption.

The sulfur-sensitive W1W sensor recorded responses between 1.53 and 2.96, with the raw tea at 1.74. Samples 1 and 3 (1:5 FTR, 2 cycles) showed lower values due to sulfur compound loss, whereas Sample 11 (1:7 FTR, 12 h, 3 cycles) peaked at 2.96 – suggesting minimal time combined with 3 cycles enhances sulfur retention.

The alkane/aliphatic-sensitive W2W sensor yielded responses of 1.18–2.13 (control: 1.37). Samples 1 and 3 demonstrated reduced values, confirming alkane/aliphatic depletion under 1:5 FTR and 2 cycles. Sample 11 showed the highest response (2.13), indicating similar retention mechanisms as sulfur compounds.

Most scented teas exceeded the control across all 10 sensors. Samples 11/12, 6/8, 10/13, and 4/7 displayed similar aroma profiles. Sensors beyond W5S/W1W/W2W showed minimal responses: W6S (1.01–1.08), W3C (0.91–1.04), and W5C (0.92–1.03). Samples 6, 9, 11, and 12 exhibited comparatively higher responses, suggesting elevated volatile compound concentrations.

As shown in Fig. S1, different scenting processes variably impacted the aroma of osmanthus black tea. Subplots a, b, and c demonstrate comparable response ranges across 12 h, 18 h, and 24 h treatments, indicating that scenting time had an insignificant impact on tea aroma profiles.

Subplots d-f revealed substantially lower responses for 1:9 FTR (Fig. S1d) versus other ratios. This likely stems from saturation of adsorption sites despite tea's inherent absorptive capacity. Excessive osmanthus flowers (>1:7 ratio) release volatiles exceeding binding thresholds, triggering competitive adsorption displacement that reduces overall aroma efficacy.

Subplots g-i demonstrated that 2-cycle processing (Fig. S1h) yielded lower volatile levels than 1 or 3 cycles. Tea's finite absorption capacity explains this phenomenon: beyond saturation, additional cycles induce volatile desorption and generate stale off-flavors. Leaf tenderness and intrinsic absorption capacity modulate outcomes (Zhao et al., 2023). Crucially, repeated cycles degrade quality through thermal damage during cyclic drying, enzymatic browning-induced leaf darkening, and cost escalation of additional cycle.

However, intermittent turning every 4 h during scenting promotes biochemical transformations. Elevated fatty aldehydes, jasmone, and benzaldehyde were documented in shaken teas versus static controls – attributable to mechanical stress-induced cell rupture that liberates membrane-bound enzymes, enhancing substrate-enzyme contact (Yin et al., 2023). This protocol not only improves tea-flower integration and heat dissipation but also amplifies aroma complexity.

### 3.4 Analysis of total volatile components

A total of 259 volatile components in 13 osmanthus black tea samples and the tea base were separated and identified using HS-SPME-GC–MS method, including 35 alcohols, 38 aldehydes, 15 acids, 21 ketones, 46 alkanes, 17 alkenes, 59 esters, 12 heterocyclics, and 17 others.

Alcohols and ketones in osmanthus black tea were 2-fold higher than in the tea base (Fig. S2), consistent with previous studies (Niu et al., 2023; Zhang et al., 2019). Increased alcohols were attributed to isophytol, absent in the tea base and sample 3, with a minimum content of 7.27 mg/kg in sample 4. Isophytol, a major alcohol in Litsea coreana Tea, has pine resin aroma and affects coagulation, calcium balance, and cardiovascular function (Ouyang et al., 2022; Wang et al., 2023).

The contents of aldehydes, nonanal, benzaldehyde and ketones increased. They may originate from the enzymatic and non-enzymatic oxidative degradation of precursors such as fatty acids, amino acids, and carotenoids (Wei et al., 2023). The average content of esters decreased by 50% compared to that in the tea base. Among them, the heterocyclic compounds in tea sample No. 13 had the least loss. The main reason for this decrease was nerolidyl acetate. It had a content of 8.33 mg/kg in the tea base but was not detected in osmanthus - scented black tea. When a large amount of osmanthus aroma components are incorporated into the tea leaves, they may compete with the original aroma components such as nerolidyl acetate, resulting in a decrease in the content of the latter in the tea leaves or making it undetectable. The total content of aroma substances in all osmanthus - scented black teas was higher than that in the black tea base (No. 0). This indicates that scenting black tea with osmanthus can increase the overall aroma content of black tea, thereby enhance the aroma of tea and improving the insufficient aroma content of summer and autumn teas.

Fig. S3 illustrates the total content and distribution of aroma components across different scenting processes, along with the effects of scenting time. Total aroma was lower at 12 h and 24 h than at 18 h, likely due to insufficient fragrance release (short time), volatile/oxidative loss of components (long time), or varying volatility/stability of different aromas. Optimizing scenting time requires consideration of tea type, flower traits, and target aroma (Chen et al., 2018; Zeng et al., 2019).

Total aroma was marginally higher when flower-to-tea ratio was 1:7 than 1:5 and 1:9. The ratio affects osmanthus black tea's temperature and humidity, influencing oxidative or degradative reactions and aroma volatility. At 1:9, tea leaves underabsorbed floral aromas; at 1:5, limited tea surface area reduced adsorption efficiency. Proper ratios facilitate optimal interaction and absorption.

Each scenting cycle enhances aroma adsorption: single-scented tea had fresh elegance, while multiple-scented tea was rich in persistence. Fig. S3 shows minor differences in volatile content across scenting cycles, with slightly higher levels (mainly alcohols) after the first cycle—attributed to strong initial absorption capacity of fresh tea leaves. Alcohols and ketones contribute floral notes, aldehydes provide citrus and green nuances, esters offer sweet coconut-like aromas, and pyrazines impart baked and caramel notes (Meng et al., 2024; Zhai et al., 2022). Osmanthus scenting enhances overall tea aroma, improving quality deficits in summer and autumn black teas.

As shown in Fig. S4, intersection analysis of all samples revealed that 52 aroma substances were retained in all osmanthus black teas. There were 10 new common aroma substances in osmanthus black teas, namely 3,7,11,15-tetramethyl-2-hexadecene, (4E,8E)-5,9,13-trimethyltetradeca-4,8,12-trienal, benzyl butyrate, n-dodecane, ionone, methyl jasmonate, n-pentadecane, hexyl hexanoate, 3,7-dimethyl-6-octene-1,2,3-triol, and 1,3,3,7-tetramethyl-8-isopropenylbicyclo[5.1.0]oct-5-en-2-one. Twelve substances disappeared during the scenting process. Tea samples 1–13 had 4, 21, 2, 5, 1, 4, 5, 1, 4, 2, 2, 1, and 2 unique compounds, respectively. Among them, sample 2 (1:9, 2 cycles, 12 h), which had the shortest scenting time and the least flower to tea ratio, contained the largest number of unique compounds (21). This may be because the short scenting time and low flower ratio prevented complete transformation and combination of aroma substances between osmanthus and black tea in a short time, and avoided excessive volatilization and degradation of aroma substances caused by long-term high temperature.

The impact of scenting time on the content of tea aroma components is highly complex, involving multiple factors such as interactions between tea and flower fragrances, chemical reactions, and the volatilization and adsorption of aroma components. For the tea samples scented for 12 h (Fig. S5A), there are 57 aroma components that belong to the original black tea. The newly generated common aroma substances include 14 volatile compounds such as benzyl butyrate and ionone. In the tea samples scented for 18 h (Fig. S5B), 69 aroma components overlap with those of the tea base. Among the newly produced aroma substances, the common ones include 15 volatile compounds such as isositsimene alcohol and benzyl butyrate. For the tea samples scented for 24 h (Fig. S5C), 67 aroma components are retained, and the common new aroma substances include 18 volatile compounds such as α-cubebene and benzyl butyrate. A further comparison of these three groups of newly generated substances is shown in Fig. S5D. The tea samples scented for 12 h have two unique compounds, namely 11-methyltricycloalkane and theaspirane. The tea samples scented for 18 h have two unique compounds, β-cyclocitral and 2,6,10,15-tetramethylheptadecane. The tea samples scented for 24 h have five unique compounds, which are α-cubebene, 1,2,3,4,4a,5,8,9,12,12a-decahydro-1,4-methylenebenzocyclodecene, n-nonanoic acid, trans-2-hexenoic acid, and chlorooctadecane.

The relationship between the content of aroma components in flower tea and different flower-to-tea ratios mainly depends on the interaction between tea leaves and flowers, as well as the balance between the release and adsorption of aroma components. For the tea-to-flower ratio of 1:5 (Fig. S6A), there are 66 original volatile substances from the tea base, and the osmanthus black tea with this ratio contains 15 identical substances such as pristane and β-cyclocitral. At a ratio of 1:7 (Fig. S6B), the osmanthus black tea shares 70 aroma components with the tea base, and among the newly generated aroma substances, there are 18 common volatile compounds including 3-methyl-tetradecane and γ-dodecalactone. When the ratio is 1:9 (Fig. S6C), the original black tea base has 59 aroma components, and after scenting, there are 16 newly generated common aroma substances such as trans-2-hexenoic acid, n-dodecane, and β-cyclocitral. It can be concluded from Fig. S6D that the ratio of 1:5 has two unique compounds, pristane and diisobutyl phthalate; the ratio of 1:7 has 4 unique compounds, namely 3-methyl-tetradecane, γ-dodecalactone, 4-[2,2,6-trimethyl-7-oxabicyclo[4.1.0]hept-1-yl]-3-buten-2-one, and arachidic acid; and the ratio of 1:9 has 3 unique compounds: 2,6,10,15-tetramethylheptadecane, 1,2,3,4,4a,5,8,9,12,12a-decahydro-1,4-methylenebenzocyclodecene, and bis(2-ethylhexyl) adipate.

The number of scenting cycles refers to the times of aroma exchange between tea leaves and fresh flowers during the production process.

Each scenting cycle is a period in which tea leaves absorb the aroma of fresh flowers. In the case of one scenting cycle (Fig. S7A), there are 72 original volatile substances from the black tea base, and the osmanthus black tea has 21 common compounds such as 2-(5-oxohexyl) cyclopentanone and xyloic acid. During the second scenting cycle (Fig. S7B), the osmanthus black tea and the tea base share 56 aroma components, with 12 common volatile compounds including β-cyclocitral and benzyl butyrate. In the third scenting cycle (Fig. S7C), there are 72 original aroma components, and there are 20 new common aroma substances such as isositsimene alcohol and benzyl butyrate. The first scenting cycle has 7 unique compounds: 2-(5-oxohexyl) cyclopentanone, xyloic acid, n-nonanoic acid, trans-2-hexenoic acid, 4-[2,2,6-trimethyl-7-oxabicyclo[4.1.0]hept-1-yl]-3-buten-2-one, theaspirane, and diisobutyl phthalate. The third scenting cycle has 4 unique compounds: hexanoic acid, 11-methyltricycloalkane, γ-dodecalactone, and 2,6,10,15-tetramethylheptadecane. However, no new substances are produced in the second scenting cycle (Fig. S7D).

### 3.5 Analysis of differential aroma compounds

**Fig. 5:**
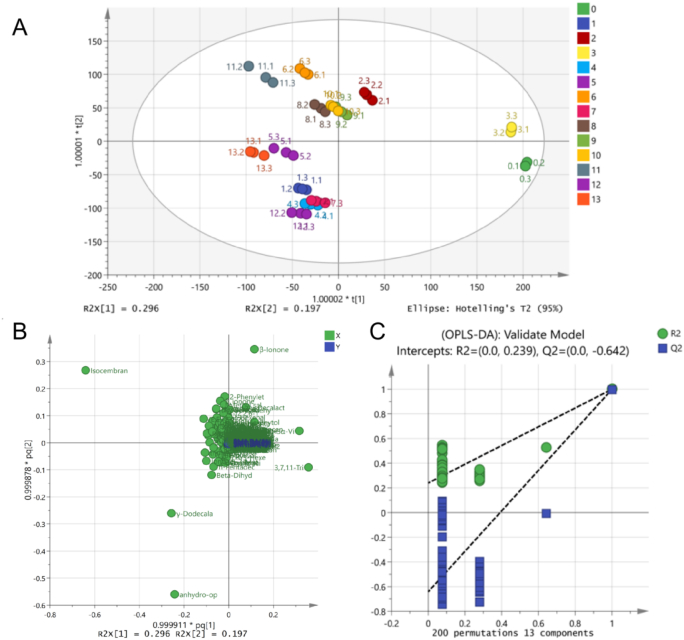
Analysis of volatile components in osmanthus black tea with different scenting processes: The dispersion point diagram (A); Load scatter plot (B); Validation of OPLS-DA model (C).

OPLS - DA combines partial least squares regression (PLSR) and orthogonal signal correction (OSC) techniques，which can distinguish samples from different groups, effectively filter out noise irrelevant to classification information, and improve the interpretability and validity of the model (Andries & Vander Heyden, 2021). Markers that can characterize sample differences are screened through VIP values, and a permutation test is used to verify the model and evaluate its accuracy (Li et al., 2023; Rivera-Perez et al., 2022). The fitting indices are R2X = 0.999, R2Y = 0.996, and Q2 = 0.992. Both R2 and Q2 are greater than 0.5 and close to 1, indicating that the model has a good fitting degree and high reliability (Z. Liu et al., 2022). A cross - validation model with 200 permutation tests was used to prove the robustness of the model (Fig. 5C). The intersection of the Q2 regression line and the vertical axis is less than zero (R2 = 0.239, Q2 = − 0.642). These indicators all prove the reliability of the OPLS - DA model, allowing for further analysis.

Principal component analysis is a data dimensionality reduction method that can convert a large number of related variables into a set of few unrelated variables (Greenacre et al., 2022). The aroma compositions of osmanthus black tea and the tea base are different,as shown in Fig. 5A. Among them, the aroma components of sample 3 (1:5, 24 h, 2 cycles) are similar to the tea base. Isositsimene alcohol, a kind of alcohol, is absent in black tea and sample 3, but accounts for a very large proportion in other teas. Samples 9 and 10 overlap, possibly because they have the same number of scenting cycles and flower-to-tea ratio. Samples 4 and 7 also have overlapping parts, which is similar to the results of the electronic nose. The abscissa represents the differences between samples, and the ordinate represents the differences within the group. The differences between each control group are significant, and the confidence of all samples is within the 95% range, indicating that the data are reliable.

The VI*P* values and P values of each aroma component were obtained through OPLS-DA analysis using SIMCA, values were sorted, and 47 differential volatile compounds were selected based on the screening criteria of VIP > 1 and *P* < 0.05. Hierarchical cluster analysis of these 47 characteristic volatile substances was visualized using a heatmap (Fig. S8). It was found at the center of the plot that all substances were divided into three categories: A, B, and C. The 14 tea samples were also classified into three groups: the first group included samples 0, 2, 3, and 6; the second group consisted of samples 1, 4, 5, 7, 12, and 13; and the third group comprised samples 8, 9, 10, and 11. Category A represents substances with relatively low content, Category B with moderate content, and Category C with overall high content. In Category A, β-ionone can be regarded as a key substance for samples 8, 9, 10, 11, 0, 2, and 3; phyt acetic acid as a key substance for samples 8 and 13; acorone as a key substance for sample 3; and nerolidyl acetate as a key substance for sample 0. In Category B, β-echinacein can be considered a key substance for samples 1, 4, 5, 7, 12, and 13, which may also serve as the basis for the clustering of the second group of teas. In Category C, isositsimene alcohol can be a key substance for all tea samples except samples 0 and 3. Additionally, linalool has been found to enhance the sweetness of sucrose solutions (Deng et al., 2024).

### 3.6 Analysis of key aroma-active compounds

The contribution of aroma components depends on their concentration and the odor threshold (Guo, Schwab, et al., 2022; Zhu et al., 2021). The importance of major aroma contributing substances is evaluated through OAV calculation (OAV = content/threshold). In this study, the thresholds of various substances were obtained from literature reports and ChemicalBook. The OAV values of aroma components in osmanthus black tea under different scenting processes were calculated. Compounds with relatively high OAV values make greater contributions to tea aroma. When OAV > 1, the compound significantly contributes to the overall aroma characteristics, and when OAV > 10, the volatile compound is identified as an important aroma component (C. Chen et al., 2022; Wang et al., 2022). After screening, 47 key aroma - contributing substances with OAV > 1, VIP > 1, and *P* < 0.05 were obtained.

As shown in Table 3, the compounds with OAV > 100 are β - ionone, γ - dodecalactone, γ - decalactone, phenylethanol, and linalool. γ - dodecalactone and phenylethanol have relatively large OAV values in 13 processes. Among them, phenylethanol is inherently present in black tea and highly retained, with little change in its content in other processes, while γ - dodecalactone and γ - decalactone are substances produced during the scenting process. γ - decalactone was only detected in tea samples 2, 3, and 6. Compounds with OAV between 10 and 100 include (E,E) - 2,4 - heptadienal, methyl salicylate, 2 - hexenal, alpha - ionone, and Δ - cadinene. Among them, α - ionone and Δ - cadinene are new substances compared to black tea. The OAV value of (E,E) - 2,4 - heptadienal in osmanthus black tea is 4 to 7 times higher than that in the tea base, indicating that scenting with osmanthus can enhance the overall fatty flavor of the tea. The sweet almond flavor of 2 - hexenal weakens during the scenting process, and methyl salicylate was not detected only in tea sample 7. Compounds with OAV between 1 and 10 are phytol, trans - α, α - 5 - trimethyl - 5 - vinyltetrahydro - 2 - furanmethanol, cis - linalool oxide, and benzaldehyde. Among them, cis - linalool oxide is not an inherent aroma substance of the original black tea. Cis - linalool oxide was not detected only in tea sample 6, but the OAV of benzaldehyde in tea sample 6 was >1, which may be due to the minimum number of scenting cycles and tea - to - flower ratio. The other three compounds all have floral aromas. Overall, the scented osmanthus black tea is mainly supplemented with γ - dodecalactone, supplemented by α - ionone.

**Table 3:** The aroma compounds of OAV > 1, VIP > 1 and P < 0.05 in osmanthus black tea with different scenting processes.

Compounds	Threshold (ug/kg)	OAV														Odor Description
		0	1	2	3	4	5	6	7	8	9	10	11	12	13	
β-ionone	6	217.73	–	352.96	386.97	–	–	–	–	390.60	345.07	444.62	590.19	–	–	floral-woody aroma, sweet fruity aroma，berry aroma
Phytol	640	2.81	–	1.95	1.93	2.19	3.09	3.25	2.91	1.18	2.56	3.30	0.06	0.02	0.06	floral aroma
γ - dodecalactone	2	–	1094.45	–	–	1169.04	1026.10	–	1064.75	577.38	498.22	659.91	969.42	1198.66	1083.14	rich fruity aroma
γ - decalactone	1.1	–	–	664.41	789.26	–	–	885.66	–	–	–	–	–	–	–	coconut butter flavor, sweet taste
(E,E) – 2,4 - heptadienal	15.4	58.31	67.40	71.74	79.17	82.84	105.85	99.02	88.85	82.41	84.00	98.89	107.13	73.24	103.97	fatty flavor
Methyl salicylate	40	9.50	11.02	10.63	10.69	10.74	13.71	16.09	–	11.99	11.94	13.59	15.58	0.54	13.81	mint flavor
Phenylethyl alcohol	0.75	1202.36	1010.53	1630.32	1505.83	1155.68	1694.68	1872.95	991.19	1363.69	1434.07	1354.75	1666.20	989.52	1539.43	honey aroma, rose floral aroma
Δ - cadinene	2	–	17.95	–	–	80.23	–	30.10	–	21.45	23.08	19.42	–	–	21.95	woody aroma


Compounds	Threshold (ug/kg)	OAV														Odor Description
		0	1	2	3	4	5	6	7	8	9	10	11	12	13	
trans – α, α – 5 - trimethyl - 5 - vinyltetrahydro - 2 – furanmethanol	60	2.84	8.68	5.35	6.16	10.00	8.14	13.55	12.44	6.82	4.90	6.18	11.77	12.93	9.36	floral aroma
cis - linalool oxide	100	–	3.72	3.55	4.01	4.37	4.58	–	4.20	3.68	3.44	4.01	5.15	4.66	4.52	earthy odor, floral aroma, woody scent
Linalool	6	77.51	86.15	110.29	106.69	81.28	114.41	130.29	113.54	108.84	98.34	108.49	148.98	106.97	106.15	citrus-floral sweetness, rose scent
2 – hexenal	30	11.02	11.10	22.32	18.97	14.27	19.64	18.21	10.94	15.33	14.48	14.17	18.45	12.76	14.40	sweet almond flavor
Benzaldehyde	750.89	0.66	0.51	0.99	0.91	0.72	0.97	1.04	0.61	0.80	0.77	0.85	0.89	0.59	0.74	almond flavor
α – ionone	10.6	–	38.10	22.84	27.14	48.63	44.10	30.92	44.53	26.97	23.84	29.13	37.59	46.39	38.57	sweet woody-floral aroma, tropical fruity scent

Note: “-” indicates not detected.

## 4 Conclusion

Osmanthus black tea was manufactured by using osmanthus and black tea made from summer and autumn tea leaves, process parameters were optimized using response surface methodology. The results showed that the most important influencing factor was scenting time. The optimal processing parameters were: a flower-to-tea ratio of 1:5.3, a scenting time of 16.6 h, and 2 scenting cycles, with an average sensory evaluation score of 92.18.

Electronic nose detection indicated that there were differences in response values mainly in the W5S, W1W, and W2S sensors (sensitive to nitrogen oxides, sulfur compounds, and aromatic hydrocarbons). Samples 6, 9, 11, and 12 showed high response values, suggesting higher contents of aroma components.

A total of 269 volatile components were identified using HS-SPME-GC–MS method, including 35 alcohols, 38 aldehydes, 15 acids, 21 ketones, 46 alkanes, 17 alkenes, 59 esters, 12 heterocyclics, and 17 others. Compared with black tea, the contents of alcohols and ketones in osmanthus black tea increased by nearly 2 times on average, while the ester content decreased by nearly half. The total content of aroma substances in all osmanthus black tea samples was higher than that in the black tea base (sample 0).

Among different scenting processes, the total aroma content was higher in samples with scenting time of 18 h. The total aroma content was slightly higher when the flower-to-tea ratio was 1:7, and the number of scenting cycles had little effect on volatile content.

Intersection analysis revealed that 56 aroma substances from black tea were retained in osmanthus black tea, and 10 new aroma substances were generated. There were 12 substances that disappeared during the scenting process, with samples 1–13 having 4, 24, 2, 5, 1, 4, 5, 1, 4, 2, 2, 2, and 3 unique compounds, respectively.

A further 49 differential substances were screened out via OPLS-DA. Cluster analysis results showed that osmanthus black tea made from the same tea base had similar key substances but different contents, which could be divided into 3 categories based on clustering. β-ionone, β-echinacein, and isositsimene alcohol served as the key substances in categories A, B, and C, respectively.

Combined with OAV analysis, 14 key aroma-contributing substances were finally screened out, including 5 newly generated substances.

## CRediT authorship contribution statement

**Haomu Liao:** Writing – original draft, Methodology, Investigation. **Xiaoyue Song:** Investigation, Formal analysis, Data curation. **Yuqin Xiong:** Software, Data curation. **Chunhua Ma:** Writing – review & editing, Project administration, Funding acquisition, Conceptualization. **Hetong Lin:** Supervision, Methodology.

## Declaration of competing interest

The authors declare that they have no known competing financial interests or personal relationships that could have appeared to influence the work reported in this paper.

## Data Availability

Data will be made available on request.

## Appendix A Supplementary data

Supplementary material Image 1
