# Supplementary material for: Optimization of processing technology and analysis of aroma components of osmanthus black tea using summer and autumn tea leaves
Source: Food Chem X. 2026 Jan 15;33:103511. doi: 10.1016/j.fochx.2026.103511 (PMC12853048; doi:10.1016/j.fochx.2026.103511)
Supplement: Supplementary file 1 — Supplementary material [file mmc1.docx]

**Optimization of Processing Technology and Analysis of Aroma Components of Osmanthus Black Tea Using Summer and Autumn Tea Leaves**

Haomu Liao ^a, b^, Feng Shi ^a, b^, Xiaoyue Song ^b^, Yuqin Xiong ^b^, Chunhua Ma ^a, *^, Hetong Lin ^b^

a College of Tea and Food Science, Wuyi University, Wuyishan, Fujian 354300, China

b College of Food Science, Fujian Agriculture and Forestry University, Fuzhou, Fujian 350002, China

* Corresponding author: Dr.Chunhua Ma

E-mail addresses: Chunhua Ma, [chma@wuyi.edu.cn](mailto:chma@wuyi.edu.cn) (C. Ma)

**Table S1 Electronic nose sensor information.**

| Sensor Name | Primary Function | Detection Range (mL/m^3^) |
| --- | --- | --- |
| W1C | Responsive to aromatic hydrocarbons & benzene derivatives | 10 |
| W5S | Sensitive to nitrogen oxides | 1 |
| W3C | High sensitivity to odor-active aromatics & ammonia compounds | 10 |
| W6S | Detects hydrogen compounds | 100 |
| W5C | Targets short-chain alkanes & aromatic VOCs | 1 |
| W1S | Selective for methylated compounds | 100 |
| W1W | Responsive to sulfur compounds & hydrocarbons | 1 |
| W2S | Optimized for alcohols, aldehydes & ketones | 100 |
| W2W | High affinity for aromatics & organic sulfides | 1 |
| W3S | Detects long-chain alkanes | 100 |


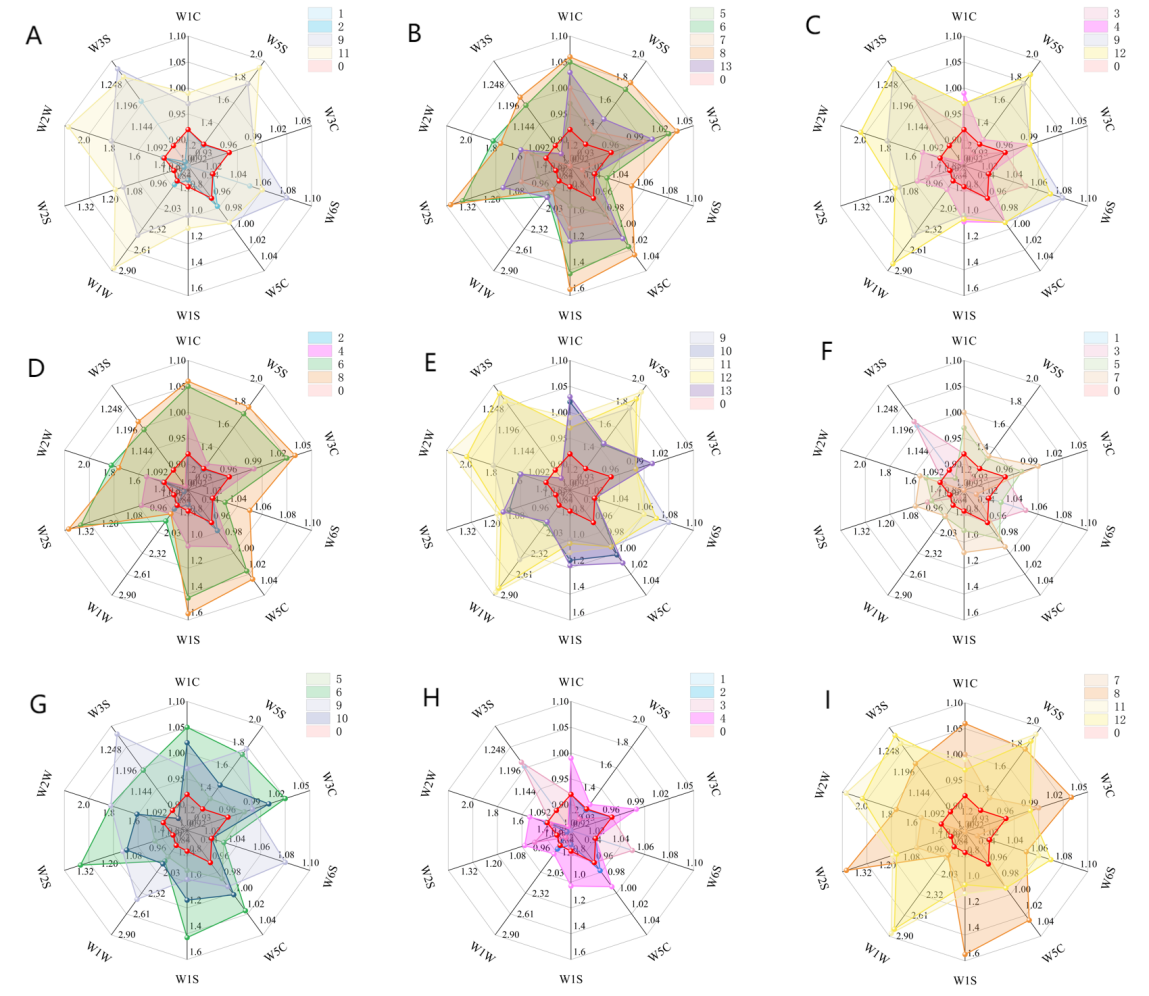


**Figure S1 Radar diagram of response value of electronic nose sensor for osmanthus black tea with different scenting processes: Scenting time was 12h, 18h, 24h (A, B, C); Flower-to-tea ratio 1:5, 1:7, 1:9 (D, E, F); Scenting cycles 1, 2, 3 (G, H, I).**


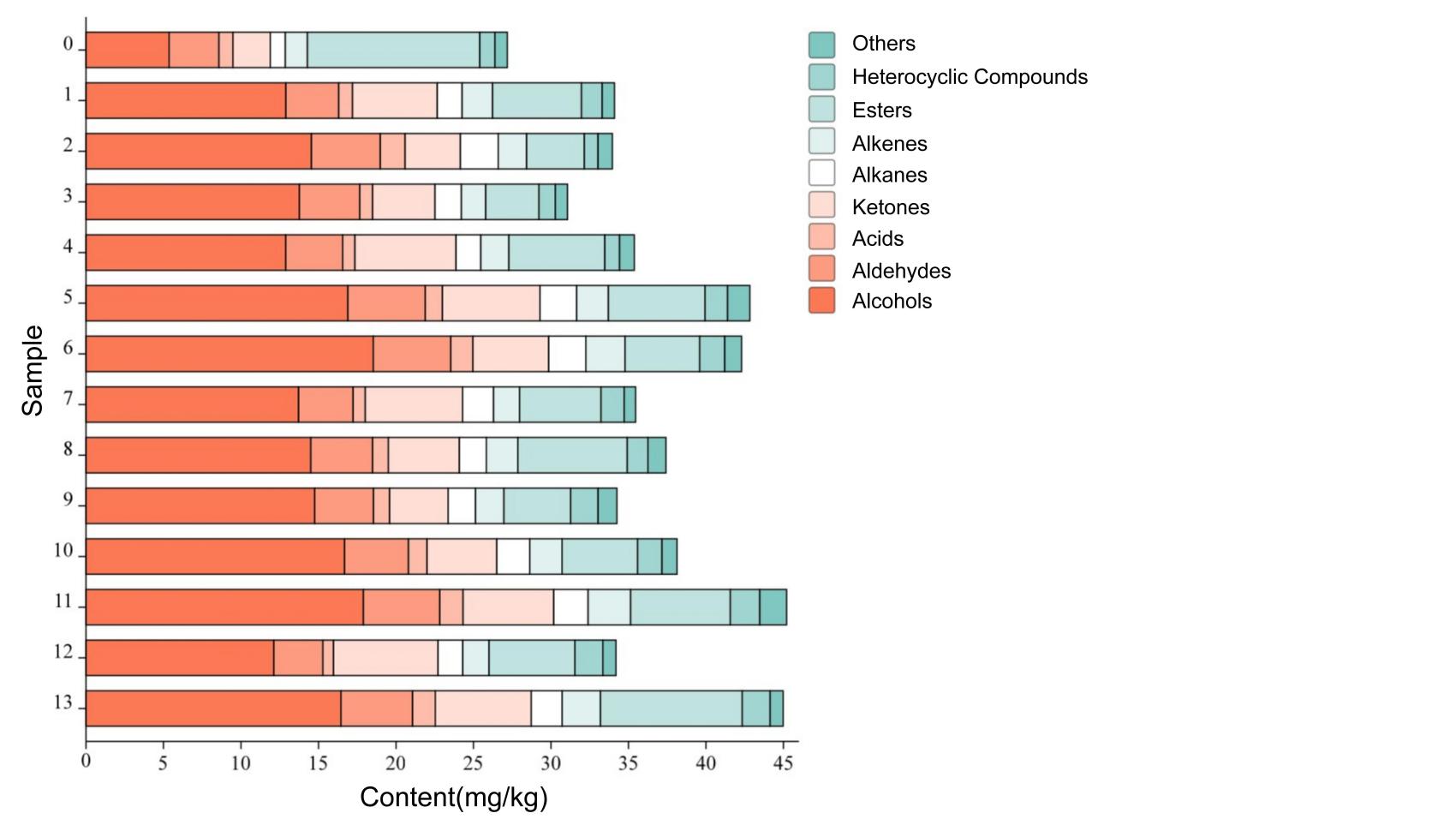


**Figure S2 Classification of aromatic compounds of osmanthus black tea by different scenting processes.**


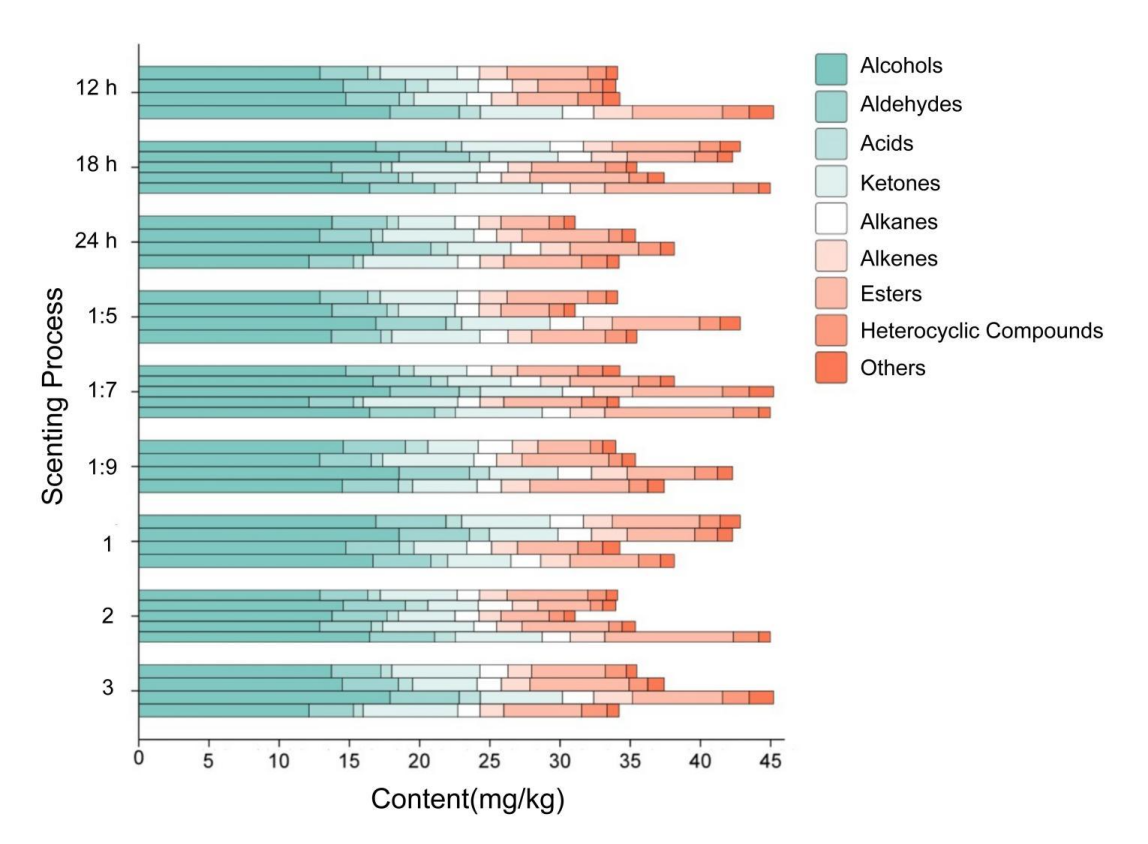


**Figure S3 Classification of aromatic compounds of osmanthus black tea by process.**


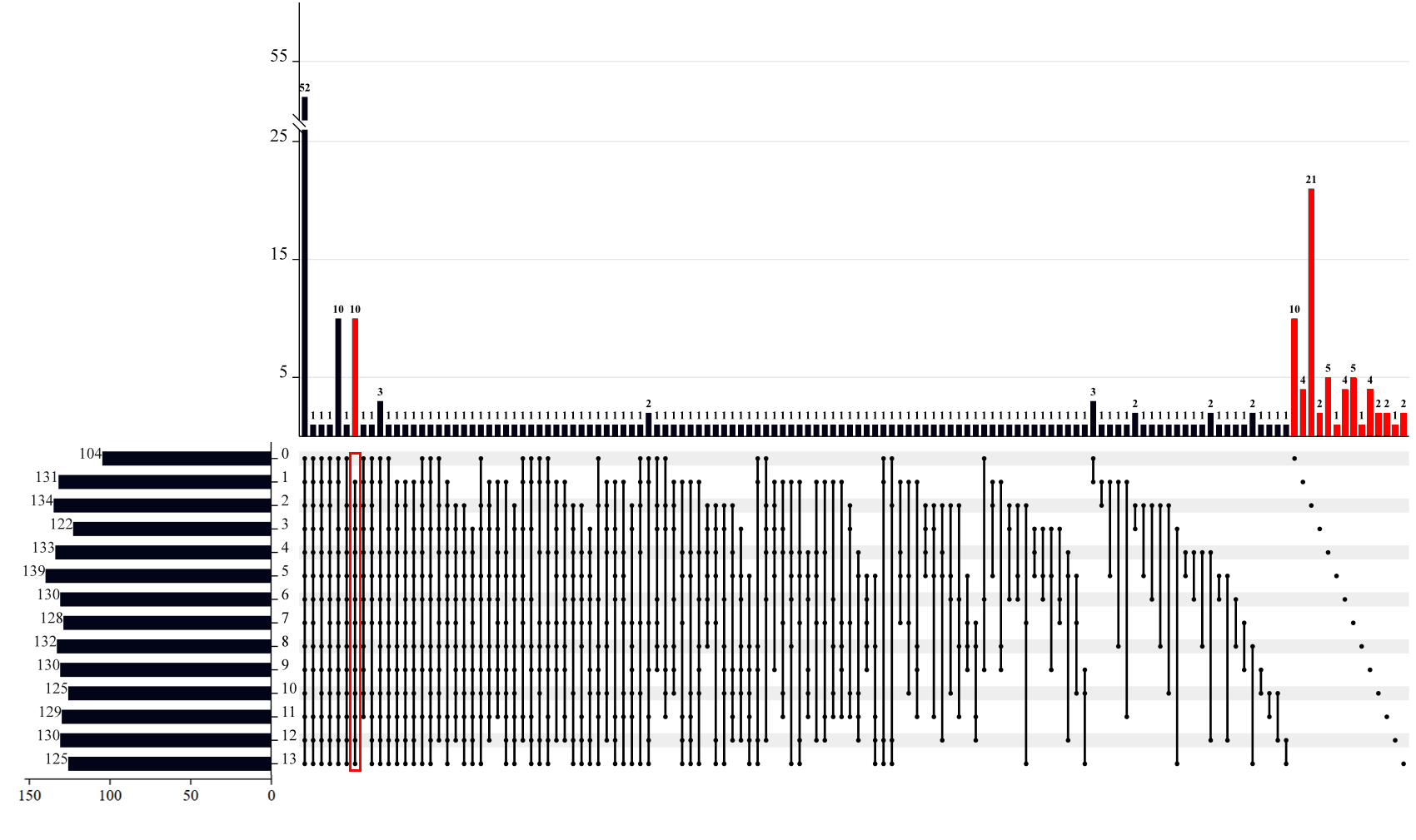


**Figure S4 Intersection analysis of different scenting processes.**


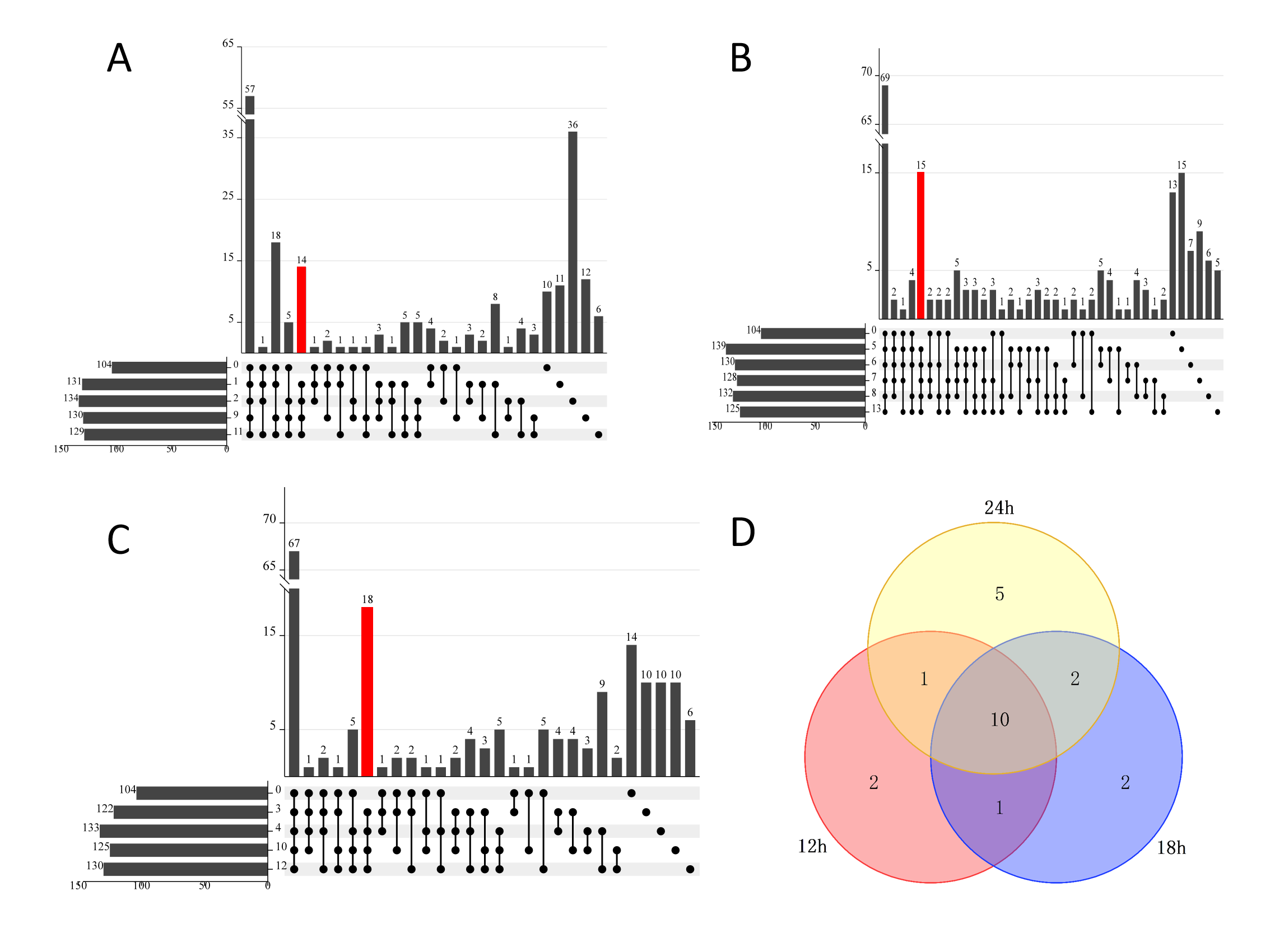


**Figure S5 Intersection analysis of different scenting time: scenting for 12 h (A); scenting for 18 h (B); scenting for 24 h (C); Differences in common substances between scenting times (D).**


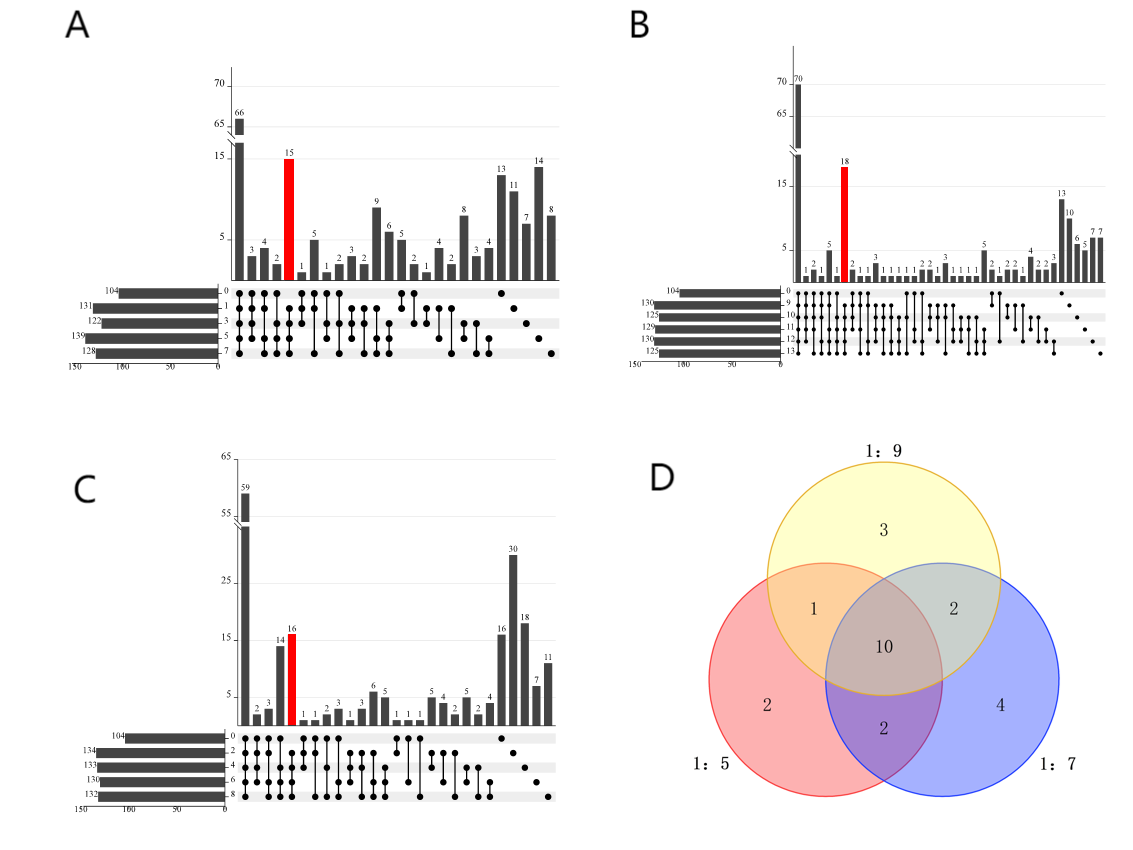


**Figure S6 Intersection analysis of different ratios of flower and tea: Flower-to-tea ratio 1:5 (A); Flower-to-tea ratio 1:7 (B); Flower-to-tea ratio 1:9 (C); Differences in common substances among different ratios of flower and tea (D).**


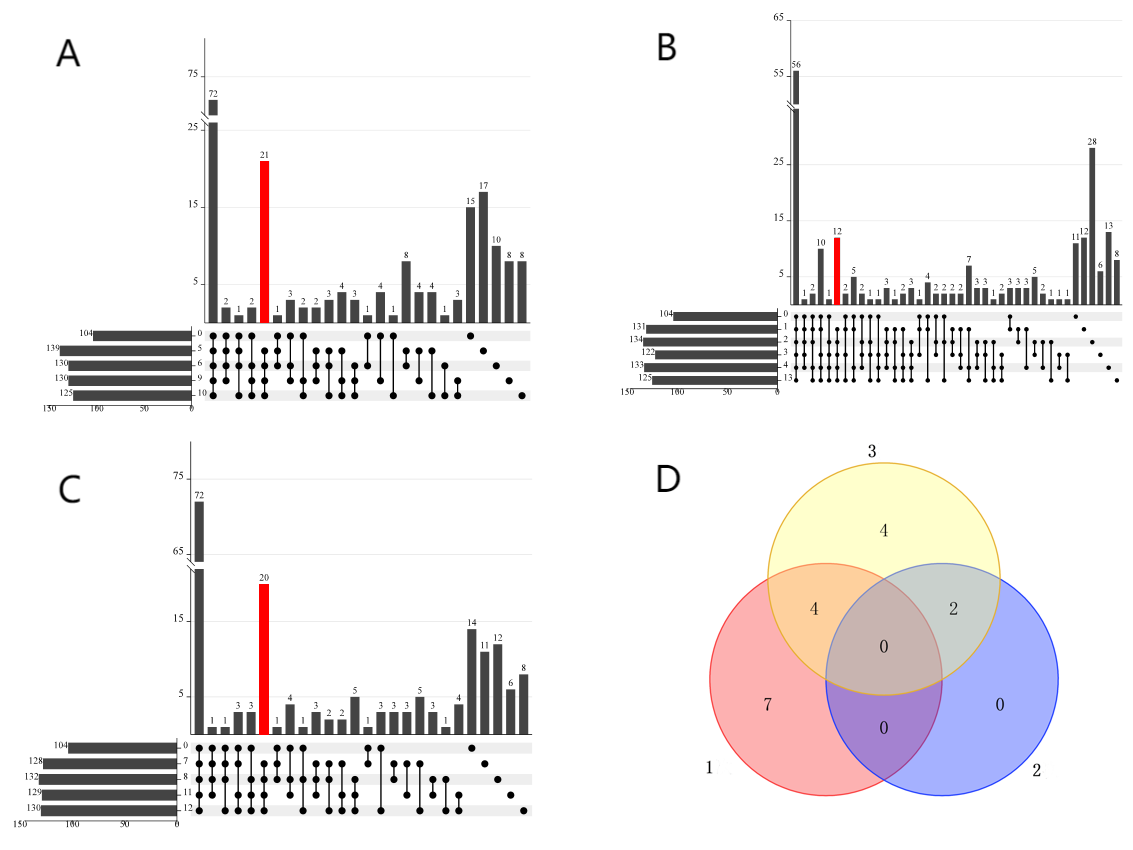


**Figure S7 Intersection analysis of different scenting frequency: scenting one (A); scenting twice (B); scenting three (C); Differences in common substances between scenting times (D).**


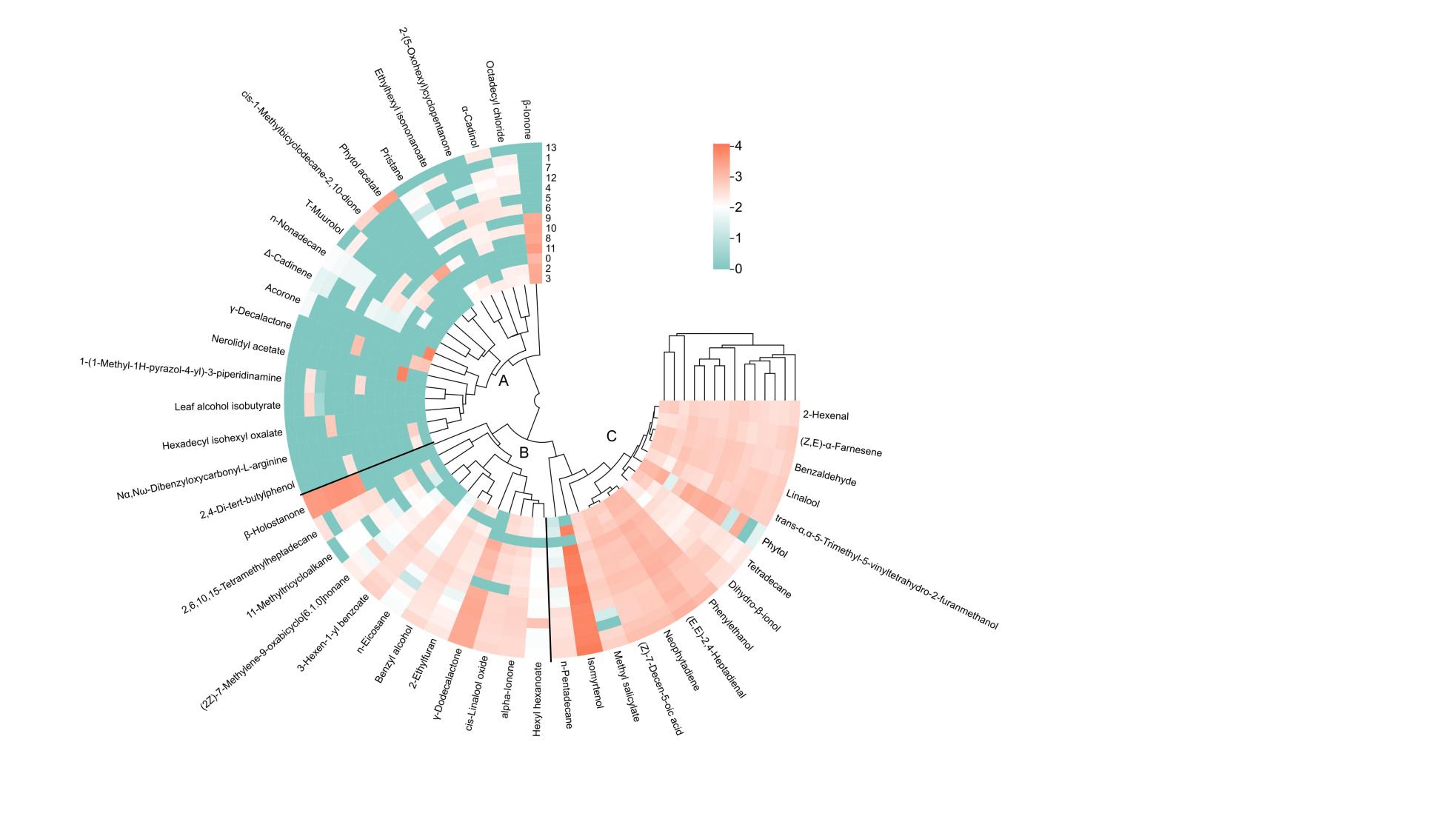


**Figure S8 Heat maps of different aroma compounds of osmanthus black tea with different scenting processes (VIP > 1, P < 0.05).**
